# Antioxidant Activity and Anti-Photoaging Effects on UVA-Irradiated Human Fibroblasts of Rosmarinic Acid Enriched Extract Prepared from *Thunbergia laurifolia* Leaves

**DOI:** 10.3390/plants10081648

**Published:** 2021-08-11

**Authors:** Thanawat Pattananandecha, Sutasinee Apichai, Jakaphun Julsrigival, Malyn Ungsurungsie, Suched Samuhasaneetoo, Pat Chulasiri, Pakakrong Kwankhao, Supaporn Pitiporn, Fumihiko Ogata, Naohito Kawasaki, Chalermpong Saenjum

**Affiliations:** 1 Cluster of Excellence on Biodiversity Based Economics and Society (B.BES-CMU), Chiang Mai University, Chiang Mai 50200, Thailand; thanawat.pdecha@gmail.com (T.P.); sutasinee.apichai@gmail.com (S.A.); jakaphun@gmail.com (J.J.); malynthai@gmail.com (M.U.); 2 Department of Pharmaceutical Sciences, Faculty of Pharmacy, Chiang Mai University, Chiang Mai 50200, Thailand; 3 Center of Excellence for Innovation in Analytical Science and Technology, Department of Chemistry (I-ANALY-S-T), Faculty of Science, Chiang Mai University, Chiang Mai 50200, Thailand; 4 Department of Food Technology, Faculty of Engineering and Industrial Technology, Silpakorn University, Nakhon Pathom 73000, Thailand; suched@yahoo.com; 5 Department of Orthopaedic, Rajavithi Hospital, Ratchathewi, Bangkok 10400, Thailand; ortho.drpat@gmail.com; 6 Chophraya Abhaibhubejhr Hospital, Prachin Anuson Rd., Tha Ngam, Mueang Prachinburi District, Prachin Buri 25000, Thailand; pakakrong2@gmail.com (P.K.); spitiporn@yahoo.com (S.P.); 7 Faculty of Pharmacy, Kindai University, 3-4-1 Kowakae, Higashi-Osaka 577-8502, Japan; ogata@phar.kindai.ac.jp (F.O.); kawasaki@phar.kindai.ac.jp (N.K.)

**Keywords:** *Thunbergia laurifolia* leaf, rosmarinic acid, antioxidants, anti-inflammatory, MMP-1

## Abstract

The current study investigated the inhibiting effect on reactive oxygen species (ROS), reactive nitrogen species (RNS), and matrix metalloproteinase-1 (MMP-1) production in a cell-based study of standardized rosmarinic acid enriched extract (SRAEE) prepared from *Thunbergia laurifolia* leaves. HPLC chromatogram revealed that rosmarinic acid is a major component in prepared SRAEE, followed by caffeic acid. SRAEE exhibited antioxidant activity both in vitro and cell-based studies. SRAEE showed scavenging effects on nitric oxide and superoxide anion and inhibition effects on lipid peroxidation in vitro. SRAEE also inhibited ROS and MMP-1 production in normal human dermal fibroblast cells induced by H_2_O_2_ and UVA, respectively, without exerted cytotoxicity. Additionally, collagen degradation was protected by SRAEE induced by UVA. Nitric oxide and inducible nitric oxide synthase (iNOS) productions were also inhibited by SRAEE in RAW264.7 mouse macrophage cells induced by combined lipopolysaccharide (LPS)-interferon-γ (IFN-γ). The results indicated that SRAEE is a potential candidate as a natural pharmaceutical active ingredient for cosmeceutical product application.

## 1. Introduction

Reactive oxygen species (ROS) and reactive nitrogen species (RNS) are highly reactive molecules that are produced in both endogenous and exogenous sources [1]. ROS and RNS are responsible for inducing DNA damage after ultraviolet (UV) radiation [2]. UV radiation is the primary cause of premature skin change, also known as photoaging. Besides this, other factors including air pollution, visible and infrared light, and endocrine factors can exacerbate photoaging. The process of photoaging also depends on skin phototype, ethnicity, and sex. In terms of pathogenesis, ultraviolet radiation is the worst causative factor in photoaging by inciting oxidative damage and pro-inflammatory pathways leading to structural changes in the skin [3]. For this reason, the prevention and treatment of these risks are important and interesting. A number of studies confirmed that the oxidation of cellular biomolecules and oxidative damage generated by UV can be prevented by antioxidant treatment [4]. 

*Thunbergia laurifolia* is a vine plant of the Acanthaceae family commonly found in tropical countries and is native to Indonesia and Malaysia. It has opposed leaves with round green stems approximately 7–18 cm long and 2.5–6.0 cm wide. Its flowers come in three colors: white, purple, or pink, are trumpet-shaped, and are approximately 8 cm long and 6–8 cm across and flower continuously throughout the year [5,6]. *T. laurifolia* has been reported as a traditional medicinal plant for the treatment of various diseases. In Thailand, the leaves and roots of *T. laurifolia* are traditionally used as an antidote for poisoning caused by ethyl alcohol, heavy metal, insecticide, and toxic drugs [7]. Currently, herbal teas, powders, and capsule formulations of *T. laurifolia* are commonly available in herbal medicine and nutraceutical markets. It has been indicated that the leaves of *T. laurifolia* are a good source of natural antioxidant phytochemicals because of the potent radical scavenging ability and inhibited electron transfer through ferric-reducing effects expressed in their extracts [8]. The extract of *T. laurifolia* has been reported to contain various biological activities—e.g., neuroprotective [9,10], hepatoprotective [11], and antimutagenic properties [12]. Rosmarinic acid and caffeic acid have been analyzed as antioxidant phytochemicals in *T. laurifolia* [10,13,14]. In addition, two iridoid glycosides namely 8-*epi*-grandifloric acid and 3′-*O*-β-glucopyranosyl-stilbericoside as well as seven known glycosides were identified from the aerial part of *T. laurifolia* [15]. Rosmarinic acid has been reported to exhibit biological activities including antimicrobial, antiviral, antioxidant, anti-inflammatory, anti-angiogenic, anti-depressant, antihyperglycemic, anti-allergic, antithrombotic, anticarcinogenic, and anti-aging [16]. Caffeic acid has been reported as a potent antioxidant and responsible for different biological activities including anti-inflammatory and anti-tumor [16,17].

In this study, it was concluded from previous literature that major antioxidant phytochemicals, namely rosmarinic acid of *T. laurifolia* might be of benefit to antioxidant activity. Hence, the objective of this study was to investigate the responses of normal human fibroblast cells and mouse macrophage cells (RAW264.7 cells) upon the treatment of *T. laurifolia* extract derived from ethanol extraction and water extraction.

## 2. Results and Discussion

### 2.1. Phytochemical Contents and In Vitro Antioxidant Activity of T. laurifolia Extracts

**Figure 1:**
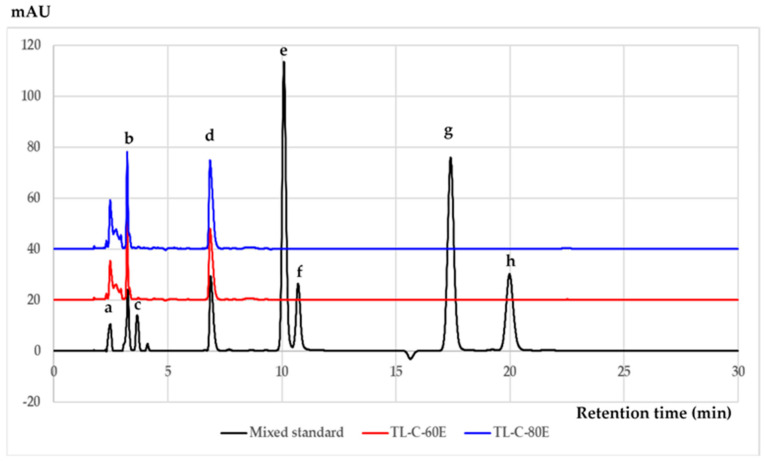
HPLC chromatograms of mixed standard, sample TL−C−60E, and sample TL−C−80E with the detection wavelength at 325 nm. The peaks indicate (a) gallic acid, (b) caffeic acid, (c) rutin, (d) rosmarinic acid, (e) luteolin, (f) quercetin, (g) apigenin, and (h) kaempferol.

The HPLC chromatograms identifying phenolic and flavonoid compounds in *T. laurifolia* extracts are shown in Figure 1. Eight standards, including gallic acid, caffeic acid, rutin, rosmarinic acid, luteolin, quercetin, apigenin, and kaempferol, were used as the mixed standard. As revealed in Figure 1, HPLC analysis method provided the well separation of all eight standards. Several analytical parameters of method validation including precision, limit of detection (LOD), and limit of quantitation (LOQ) were examined as shown in Table 1. The calibration curves were linear in the range studied from 5–100 μg/mL with correlation coefficients (R^2^) of more than 0.9992 for each compound. LOD and LOQ were determined as 3 and 10 standard deviations of the blank signal (*n* = 7). The LOD and LOQ values ranged from 0.11–0.40 μg/mL and 0.36–1.35 μg/mL indicating sufficient sensitivity of analysis method. Additionally, intra-day and inter-day precisions of retention time and peak area expressed as RSD were less than 2.0%. The sample chromatograms showed *T. laurifolia* extract contains rosmarinic acid as a major component, followed by caffeic acid. The phytochemical contents of rosmarinic acid and caffeic acid of all *T. laurifolia* extracts are displayed in Table 2.

The results demonstrated that extracted samples with chlorophyll separation, namely TL−C−W, TL−C−60E, and TL−C−80E had significantly higher phytochemical contents than that of samples without chlorophyll separation (*p* < 0.05). Ethanolic extract samples also had significantly higher phytochemical contents than the water extract sample (*p* < 0.05). The *T. laurifolia* extracts, TL−C−80E, significantly contained the highest amount of rosmarinic acid (*p* < 0.05) and both TL−C−80E and TL−C−60E also contained the highest amount of caffeic acid. Rosmarinic acid is an ester of caffeic acid and 3,4-dihydroxyphenyllactic acid [16]. It has a variety of interesting biological activities such as astringent, antioxidative activity, antimutagen, antibacterial, and antiviral effects [17]. Caffeic acid is a potent antioxidant and has different biological activities including anti-inflammatory and anti-tumor [16,17]. The constituents of *T. laurifolia* extracts have been reported in various studies such as rosmarinic acid, caffeic acid, rutin, pyrogallol, catechin, apigenin, isoquercetin, and quercetin using different identification methods including HPLC, nuclear magnetic resonance spectroscopy (NMR), and liquid chromatography-mass spectrometry (LC−MS) [10,13,18,19,20].

The prepared *T. laurifolia* extracts were determined for antioxidant activity in both ROS and RNS systems using in vitro and cell-based studies. The results from in vitro antioxidant activity measurements, including scavenging effects on nitric oxide and superoxide anion, and inhibition on lipid peroxidation are shown in Table 3.

Chlorophyll separation significantly (*p* < 0.05) affects the in vitro antioxidant activity of *T. laurifolia* extracts. Ethanolic extracts of *T. laurifolia* prepared from chlorophyll separated raw material (TL−C) exhibited a greater hydrophilic antioxidant (nitric oxide and superoxide anion scavenging) and the inhibition effect on lipid peroxidation than that of ethanolic extracts prepared from raw material without chlorophyll separation (TL). Moreover, ethanolic extracts exerted a higher in vitro antioxidant activity comparable to the water extracts. The results indicated that ethanolic extracts contain both hydrophilic and lipophilic antioxidants, which correspond to the studies reported previously by Suwanchaikasem et al. [7], who isolated major antioxidant compounds, namely, rosmarinic acid from *T. laurifolia*, using antioxidant-guided isolation by scavenging effect on 1,1-diphenyl-2-picrylhydrazy radical (DPPH). Rosmarinic acid also prevented lipid peroxidation and interacted with lipids [15]. The results indicated the correlation between phytochemical content and in vitro antioxidant activity. TL−C−60E and TL−C−80E exhibited the highest in vitro antioxidant activity through inhibition effects on lipid peroxidation and scavenging effects on nitric oxide and superoxide anion corresponding to the highest rosmarinic acid content. The results demonstrated that the separation of chlorophyll resulted in the higher phytochemical contents and in vitro antioxidant activity of *T. laurifolia* extracts. These may be due to the portion of more potent antioxidant contents being higher after chlorophyll was removed. Phaisan et al. [21] reported that chlorophyll removal from plant extracts improved phenolic and flavonoid contents and also antioxidant activity. The factors including plant location, extraction methods, and leaf stage also affected the difference of the phytochemical contents and their antioxidant activity [20,22,23].

### 2.2. Antioxidant and Biological Activities in Cell-Based Studies

**Figure 2:**
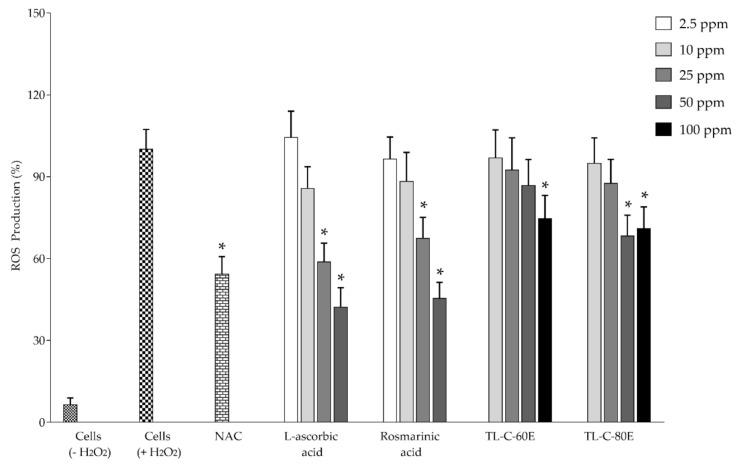
Effects of SRAEE on H_2_O_2_-induced ROS in NHDF cells. N-acetylcysteine (NAC, 80 μM), L-ascorbic acid (250 mM), and rosmarinic acid (150 mM) were used as positive controls. Data represents the mean ± SD of three independent experiments. * Indicate a significant difference of experimental samples versus treated cells with H_2_O_2_ induction (*p* < 0.05).

**Figure 3:**
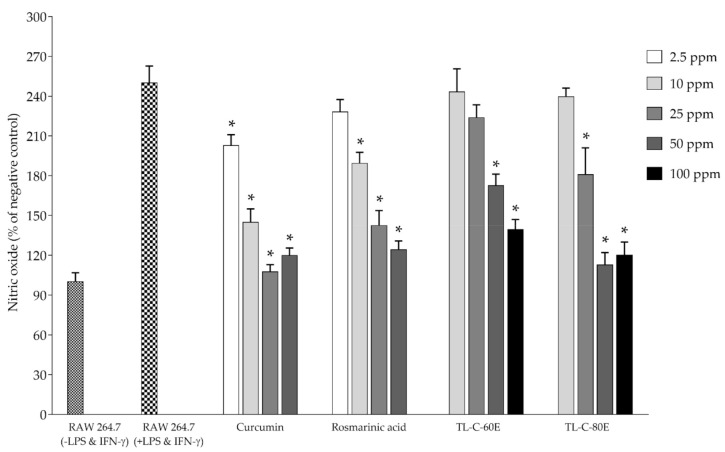
Inhibition effect of SRAEE on nitric oxide production. Data represents the mean ± SD of three independent experiments. * Indicate a significant difference of experimental samples versus treated cells with combined LPS and IFN-γ induction (*p* < 0.05).

**Figure 4:**
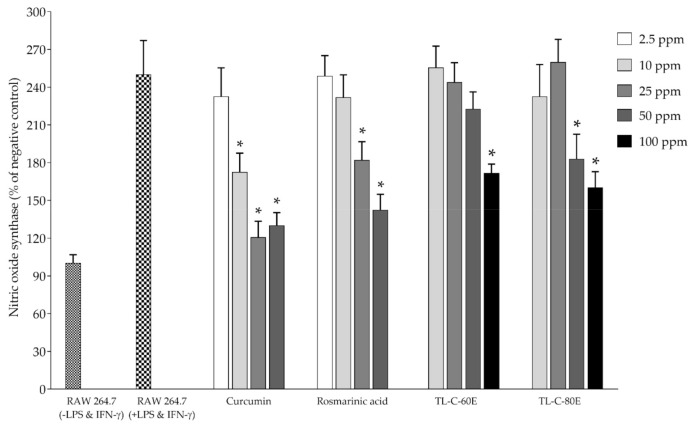
Inhibition effect of SRAEE on inducible nitric oxide synthase production. Data represents the mean ± SD of three independent experiments. * Indicate a significant difference of experimental samples versus treated cells with combined LPS and IFN-γ induction (*p* < 0.05).

A high amount of rosmarinic acid content and in vitro antioxidant activity, TL−C−60E and TL−C−80E, were selected to investigate biological activities, including inhibition effect on nitric oxide and iNOS production in RAW264.7 mouse macrophage cells. The inhibition effects on ROS production in normal human skin fibroblast (NDHF) were also determined. Before investigation, the antioxidant and biological activities of TL−C−60E and TL−C−80E, the cytotoxicity to RAW264.7, and normal human skin fibroblast (NDHF) cells were also determined using a cell proliferation reagent. The results indicate that the tested concentrations ranging from 10−100 ppm did not exert cytotoxicity to both cells. Standardized rosmarinic acid enriched extract (SRAEE) prepared from *T. laurifolia* leaf, including 50 and 100 ppm of TL−C−80E and 100 ppm of TL−C−60E, significantly exhibited an antioxidant activity through an inhibitory effect on ROS production in NHDF cells induced by H_2_O_2_ when compared to the H_2_O_2_-induced NHDF cells (*p* < 0.05) (Figure 2). SRAEE also prevented RNS production in RAW264.7 cells induced by combined LPS-IFN-*γ*. TL−C−60E at 50 and 100 ppm and TL−C−80E at 25, 50, and 100 ppm significantly inhibited nitric oxide production. Additionally, 50 and 100 ppm of TL−C−80E and 100 ppm of TL−C−60E significantly inhibited iNOS production compared to the combined LPS-IFN-*γ* induced RAW264.7 cells (*p* < 0.05) as shown in Figure 3 and Figure 4, respectively, without exerted cytotoxicity in the concentration lower than 100 ppm. TL−C−80E, which contains a higher amount of rosmarinic acid, exhibited a stronger activity in cell-based studies comparable to TL−C−60E. 

The results indicate that SRAEE prepared from *T. laurifolia* leaf was concentration-dependent in exerting antioxidant activity by inhibiting ROS production in NHDF cells induced by H_2_O_2_ and RNS production in RAW264.7 cells induced by combined LPS and IFN-γ without exerting cytotoxicity at the tested concentrations. Oonsivilai et al. [8] reported that *T. laurifolia* leaf extract showed weak or no cytotoxic activity against BHK(21)C123 and L929 normal cell, HepG2, and Caco-2 cells using MTT assay. *T. laurifolia* leaf extract also had no effect when administered at 20–2000 mg/kg/day in Wistar rats during chronic toxicity tests [24]. Rosmarinic acid in *Prunella vulgaris* ethanolic extract (10 µg/mL) was reported to inhibit LPS-induced prostaglandin E2 (PGE2) and nitric oxide production in RAW264.7 cells [25]. *T. laurifolia* extract containing rosmarinic acid, caffeic acid, rutin, and pyrogallol has been shown to reduce nitric oxide production and increase cell proliferation using RAW264.7 cells [10]. Recent studies by Mairuae et al. [26] demonstrated that rosmarinic acid extracted from *T. laurifolia* not only exerted anti-oxidative activity by suppressing ROS production but also suppressed nitric oxide production in LPS-stimulated BV2 cells. Nitric oxide plays an important role in inflammatory mediators produced by three isoforms of NOS, namely neuronal NOS, iNOS, and endothelial NOS, under physiological and pathophysiological conditions, and it also acts as a crucial mediator during the inflammatory process. The increasing nitric oxide production and iNOS expression lead to the important cytotoxic function of LPS-stimulated macrophages [27,28].

**Figure 5:**
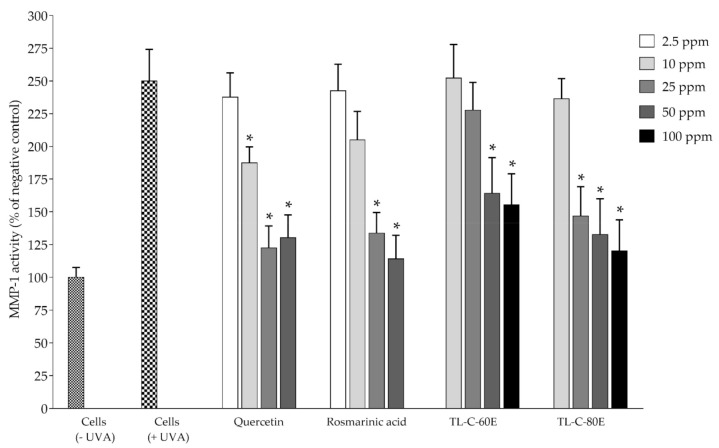
Inhibition effect on MMP-1 production. Data represents the mean ± SD of three independent experiments. * Indicate a significant difference of experimental samples versus treated cells with UVA irradiation (*p* < 0.05).

**Figure 6:**
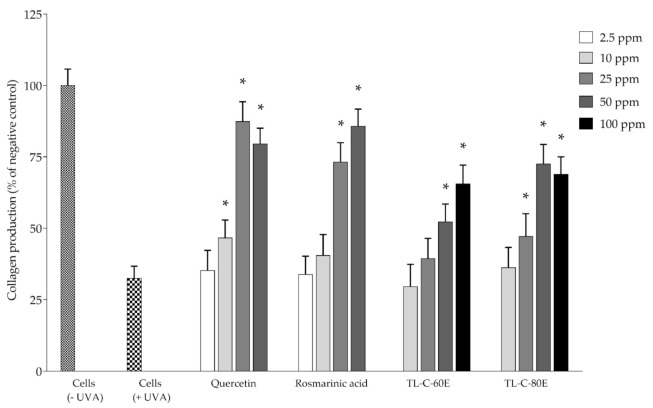
Protective effect on collagen degradation. Data represents the mean ± SD of three independent experiments. * Indicate a significant difference of experimental samples versus treated cells with UVA irradiation (*p* < 0.05).

Matrix metalloproteinase-1 (MMP-1) is the enzyme involved in the degradation of collagen types I, II, and III [29]. In normal physical conditions, the amount of MMPs is relatively low. However, MMPs can be stimulated by exposure to UV rays in sunlight both in vitro cultured cells and in vivo [29,30]. TL−C−60E and TL−C−80E were selected to investigate anti-photoaging effects on UVA-irradiated NHDF cells. The levels of MMP-1 and collagen were analyzed and are shown in Figure 5 and Figure 6, respectively. The results revealed that TL−C−60E at 50 and 100 ppm and TL−C−80E at 25, 50, and 100 ppm, significantly inhibited MMP-1 production and reduced collagen degradation by 47.1 ± 7.97% and 72.5 ± 6.83% (*p* < 0.05), respectively, when compared to UVA-induced NHDF cells. These results demonstrated that SRAEE is a potent antioxidant that not only protects against MMP-1 production but also prevents collagen degradation in UVA-induced NHDF cells. The previous research indicated that *Rosmarinus officinalis* L. extract containing rosmarinic acid, carnosol, and carnosic acid had been shown to protect against UV-induced MMP-1 in human dermal fibroblasts and reconstructed skin [31]. Rosmarinic acid was also shown to inhibit MMP-2 and MMP-13 activity [32,33,34] and was recently reported to protect human fibroblasts from damage and stimulates collagen biosynthesis [35]. Our results indicated a potential candidate of SRAEE prepared from dechlorophyll of *T. laurifolia* leaf, which exhibited antioxidant activity and anti-photoaging effects on UVA-irradiated NHDF cells as a natural active pharmaceutical ingredient for cosmeceutical product application.

## 3. Materials and Methods

### 3.1. Chemicals and Reagents

Gallic acid, caffeic acid, rutin, rosmarinic acid, luteolin, quercetin, apigenin, and kaempferol were obtained from Sigma Chemical Co. (St. Louis, MO, USA). All chemicals and solvents used were either standard, analytical, or HPLC grade and were purchased commercially from Sigma Chemical Co., Ltd. (St. Louis, MO, USA), Merck Co., Ltd. (Kenilworth, NJ, USA), or Fluka Chemical Co. (Buchs, Switzerland). All chemicals and reagents used in the cell-based study were purchased from Invitrogen (Waltham, MA, USA) and Roche (Mannheim, Germany).

### 3.2. Thunbergia laurifolia Leaf Sample and Preparation of Rosmarinic Acid-Enriched Extract (RAEE)

Experiments were conducted on *T. laurifolia* leaf harvested from an organic farm in Chaophraya Abhaibhubejhr hospital, a well-known Thai herbal medicine hospital located in Prachin Buri province, Thailand in November 2018. The *T. laurifolia* leaf was dried at 50 °C in a hot air oven for 24 h and ground into a coarse powder. The raw material of *T. laurifolia* leaf was divided into 2 parts: without (TL) and with (TL−C) pretreated with chlorophyll separation by acetone extraction. The powder of each raw material was separately extracted with deionized water at 100 °C (W), 60% (60E), and 80% ethanol (80E) at 80 °C using an incubator shaker at 120 rpm for 2 h. The solutions were collected, filtrated, and removed from the solvent under reduced pressure and then vacuum dried to obtain the water extract (TL−W and TL−C−W), 60% ethanolic extract (TL−60E and TL−C−60E), and 80% ethanolic extract (TL−80E and TL−C−80E).

### 3.3. Chromatographic Analysis of Phenolic and Flavonoid Compounds

Gallic acid, caffeic acid, rutin, rosmarinic acid, luteolin, quercetin, apigenin, and kaempferol were analyzed by reverse-phase HPLC using an Agilent 1200 equipped with a multi-wavelength detector. The detection wavelength was set at 325 nm. The assay was performed using a Symmetry Shield RP18 column (4.6 mm × 250 mm, 5 µm particle diameters, Waters Co., Ltd., Milford, MA, USA) and 25% acetonitrile in 0.1% acetic acid and deionized water was used for the mobile phase at the flow rate of 1.0 mL/min. The column temperature was set to 25 °C with an injected sample volume of 10 µL. The method was validated following EURACHEM guideline. The intra-day precision (repeatability) and inter-day precision (within-laboratory reproducibility: measurements were performed on three different days) were checked. Determined validation parameters were linearity, limit of detection (LOD), and limit of quantitation (LOQ). Linearity was performed using mixed standard solutions at six concentrations of each compound.

### 3.4. Determination of In Vitro Antioxidant Activity

#### 3.4.1. Scavenging Effects on Nitric Oxide

The scavenging activity of tested samples on nitric oxide was determined using the improved method of Sreejayan and Rao [36] and Kidarn et al. [37]. Briefly, 800 μL of sodium nitroprusside in phosphate buffer saline (PBS) pH 7.4 was mixed with 200 μL of various concentrations of tested samples in the final concentration range from 10–200 μg/mL or the positive controls, rosmarinic acid and curcumin. The mixture solutions were incubated at 37 °C for 150 min. Then, 100 μL of Griess reagent was prepared by equal mixing of 0.1% (*w*/*v*) naphthylethylenediamine dihydrochloride (NEDA) with 1% (*w*/*v*) sulfanilamide in phosphoric acid was mixed with 150 μL of the mixture solutions in 96-wells plate. The absorbance of the formed color after 5 min of reaction time was measured at 540 nm spectrophotometry. The results were calculated and expressed as 50% inhibition concentration (IC_50_).

#### 3.4.2. Scavenging Effects on Superoxide Anion

The scavenging activity of tested samples on superoxide anion was investigated using the improved method of Yangping et al. [38] and Saenjum et al. [39]. Briefly, the mixture solution consisted of β-nicotinamide adenine dinucleotide, nitroblue tetrazolium, ethylenediaminetetraacetic acid, and various concentrations of the tested in PBS pH 7.4, rosmarinic acid and L-ascorbic acid used as the positive controls. The reaction was initiated by adding 25 µL of phenazine methosulphate and then incubated for 5 min with light protection. Then, the absorbance was immediately measured at 560 nm spectrophotometry. All the samples were tested in triplicate. The results were calculated and expressed as IC_50_.

#### 3.4.3. Inhibition Effect on Lipid Peroxidation

The inhibition effect on lipid peroxidation was investigated using the improved method by Saenjum et al. [39]. Briefly, the linoleic acid emulsion was prepared by linoleic acid and tween 20 in Tris-HCl pH 7.5. The reaction mixture consisted of a linoleic acid emulsion, L-ascorbic acid, and various concentrations of the tested samples. Rosmarinic acid and α-tocopherol were used as the positive controls. Fe_2_SO_4_·7H_2_O was added to initiate the reaction and then incubated at 37 °C for 30 min. Trichloroacetic acid and 1% (*v*/*v*) of thiobarbituric acid in 50 mM NaOH were added to terminate the reaction and then heated at 100 °C for 10 min. Finally, the formed color solutions were spectrophotometrically measured at 532 nm. All the samples were tested in triplicate. The percentage of linoleic acid peroxidation inhibition was calculated and expressed as IC_50_.

### 3.5. Determination of Antioxidant and Biological Activities in Cell-Based Studies

The *T. laurifolia* leaf extracts that exhibited a potent in vitro antioxidant activity were selected to investigate antioxidant and biological activities in the cell-based study.

#### 3.5.1. Determination on Inhibition Effect on Intracellular ROS Production

The inhibitory effect of the selected samples on intracellular ROS production was determined using the improved dichloro-dihydro-fluorescein diacetate (DCFH−DA) method of Phromnoi et al. [40]. An amount of 1 × 10^6^ cells/mL of normal human dermal fibroblast (NHDF) was plated into a 96-well culture plate for 12 h. Cells were then pre-treated with tested samples in the concentrations of 10–100 µg/mL for 12 h followed by treatment with 5 mM hydrogen peroxide (H_2_O_2_) for 30 min to induce ROS production. A total of 40 µM of DCFH−DA solution was then added to each well-plate and incubated at 37 °C with 5% CO_2_ for 30 min. The green fluorescence intensity was measured using the excitation wavelength at 480 nm and emission wavelengths at 525 nm. N-acetyl cysteine, rosmarinic acid, and L-ascorbic acid were used as positive controls.

#### 3.5.2. Determination of the Effect on Nitric Oxide and Inducible Nitric Oxide Synthase (iNOS) Production

The inhibition effect on nitric oxide and iNOS production in mouse macrophage cells (RAW264.7) was determined using the method of Hong et al. [41], Hu et al. [42] and Sirithunyalug et al. [43]. Briefly, RAW264.7 cells were cultured in 24-well plates with Dulbecco’s Modified Eagle’s Medium supplemented with 10% fetal bovine serum, 100 units/mL penicillin, and 100 μg/mL of streptomycin then pre-incubated at 37 °C and 5% CO_2_ for 12 h. The cultured cells were then removed and given a fresh medium containing final concentrations in the range of 10–100 μg/mL of tested samples. After 12 h of the incubation period, the LPS and IFN-γ in the final concentrations of 2 ng/mL and 50 pg/mL, respectively, were added and incubated at 37 °C with 5% CO_2_ for 72 h. The NO level was measured in the supernatants of the cultured medium using the Griess reagent at 540 nm spectrophotometrically and calculated using a calibration curve of potassium nitrite. Fresh cultured medium was used as a blank. Furthermore, CelLytic^TM^ M Cell Lysis Buffer (Sigma, C2978, MO, USA) was used to prepare cell lysate for iNOS and protein analysis. The iNOS levels in cell lysates were determined using a commercially available mouse iNOS ELISA kit (CSB-E08326M, Cusabio Biotech, Co., Ltd., Houston, TX, USA). Rosmarinic acid and curcumin, naturally anti-inflammatory and antioxidant compounds, were used as positive controls. Cell viability and protein concentrations of control samples and those stimulated with combined LPS and IFN-γ for 72 h were assayed using cell viability reagent (PrestoBlue^TM^, Invitrogen, MA, USA) and Bradford reagent, respectively [18].

#### 3.5.3. Determination on Matrix Metalloproteinase-1 (MMP-1) and Collagen Production

The determination of MMP-1 was modified from the method of Huang et al. [44]. NHDF cells were cultured in growth medium FGMTM-2 BulletKitTM supplemented with FBM^®^ (fibroblast basal medium) and GA-1000 (gentamicin sulfate amphotericin-B), 0.5 mL insulin (recombinant human), and rhFGF-B (r-human fibroblast growth factor-B) at 37 °C containing 5% CO_2_. An amount of 1 × 10^5^ cells of NHDF were plated in a 24-well plate/well and incubated for 12 h. NHDF cells were then pretreated with tested samples at a concentration ranging from 10–100 μg/mL in a complete growth medium for 24 h after which the medium was removed and cells were washed twice by PBS (Invitrogen, Waltham, MA, USA). Hank’s balanced salt solution (Sigma-Aldrich, Co., St. Louis, MO, USA) was added and the cells were irradiated with UVA at 8 J/cm^2^. Tested cells without exposure to UVA were used as a control. After irradiation, HBSS was removed, and a fresh complete growth medium was added in the presence of the indicated concentration of the tested sample and incubated for 24 h. Furthermore, CelLytic^TM^ M Cell Lysis Buffer (Sigma, C2978) was used to prepare cell lysate for MMP-1 and collagen measurement. Rosmarinic acid and quercetin were used as positive controls. The MMP-1 levels in cell lysates were determined using a commercially available human MMP-1 ELISA kit (RayBio^®^ ELH-MMP1–1, RayBiotech Life, Inc., GA, USA). Cell viability and protein concentrations were measured using the methods described previously in 3.5.2. Additionally, the collagen levels and the hydroxyproline was measured in the lysate by RP-HPLC, according to the methods described previously by Ruangsuriya et al. [45]. Briefly, the lysates were subjected to acid hydrolysis using 6M HCl and measured to obtain hydroxyproline by RP-HPLC. The column used in this analysis was the Kinetex^®^ C18 column, 250 × 4.6 mm in diameter (Phenomenex Co., Ltd., CA, USA). The mobile phase consisted of 100 mM sodium acetate buffer and acetonitrile (93:7) with a flow rate of 0.3 mL/min and the wavelength for detection was at 495 nm. Different concentrations of the standard hydroxyproline were used to set a standard curve and the amounts of hydroxyproline in all samples were calculated accordingly.

### 3.6. Statistical Analysis

SPSS software (version 17.00) was used to statistically analyze all the data. A one-way ANOVA was used for finding any significant difference between treatments, *p* < 0.05 was considered to be significant and further significance between groups was analyzed using a Duncan post hoc test. Results are presented as the mean ± standard deviation of 3 independent experiments.

## 4. Conclusions

Standardized rosmarinic acid enriched extract (SRAEE) prepared from *T. laurifolia* leaf exhibited scavenging effects on nitric oxide and superoxide anion and inhibition effects on lipid peroxidation in vitro. Additionally, SRAEE inhibited ROS and MMP-1 production in NHDF cells induced by H_2_O_2_ and UVA, respectively, without exerted cytotoxicity. Moreover, collagen degradation was protected by SRAEE in NHDF cells-induced by UVA. Nitric oxide and inducible nitric oxide synthase (iNOS) productions were inhibited by SRAEE in RAW264.7 mouse macrophage cells induced by combined LPS-IFN-γ. The results indicated that SRAEE is a potential candidate as a natural pharmaceutical active ingredient for cosmeceutical product application.

## Figures and Tables

**Table 1:** Performance characteristics evaluated during method validation.

Compounds	Precision (% RSD)				Linear Range (μg/mL)	Correlation Coefficient	LOD (μg/mL)	LOQ (μg/mL)
	Retention Time		Peak Area					
	Intra-Day	Inter-Day	Intra-Day	Inter-Day				
Gallic acid	1.09	1.28	0.85	0.97	5–100	0.9998	0.24	0.83
Caffeic acid	1.02	1.15	0.89	1.02	5–100	0.9992	0.40	1.35
Rutin	1.25	1.42	1.06	1.13	10–100	0.9994	0.30	1.01
Rosmarinic acid	1.01	1.18	0.81	0.92	5–100	0.9998	0.19	0.62
Luteolin	0.93	1.03	0.78	0.90	5–100	0.9998	0.11	0.36
Quercetin	1.11	1.32	0.98	1.09	5–75	0.9997	0.13	0.45
Apigenin	0.75	0.92	0.62	0.74	5–100	0.9995	0.25	0.82
Kaempferol	0.65	0.78	0.58	0.65	10–100	0.9996	0.33	1.07

**Table 2:** Rosmarinic acid and caffeic acid contents in *T. laurifolia* extract.

Samples	Phytochemical Content (mg/g Extract)	
	Rosmarinic Acid	Caffeic Acid
TL−W	147.3 ± 10.4 ^f^	120.8 ± 13.4 ^e^
TL−60E	177.6 ± 12.3 ^d,e^	158.2 ± 10.7 ^c,d^
TL−80E	183.2 ± 10.6 ^d^	161.9 ± 12.6 ^b,c^
TL−C−W	221.8 ± 17.7 ^c^	147.2 ± 11.4 ^d^
TL−C−60E	260.5 ± 16.6 ^b^	186.8 ± 12.7 ^a^
TL−C−80E	292.3 ± 18.3 ^a^	195.4 ± 16.5 ^a^

All values are expressed as mean ± standard deviation (*n* = 3). Different letters in each column indicate a significant difference (*p* < 0.05).

**Table 3:** IC_50_ on nitric oxide and superoxide anion scavenging activities and inhibition effect on lipid peroxidation.

Samples/Positive Control		IC_50_ (ppm)	
	Nitric Oxide	Superoxide Anion	Lipid Peroxidation
TL−W	43.84 ± 1.87 ^f,g^	35.55 ± 0.82 ^f^	72.28 ± 3.23 ^g,h^
TL−60E	38.57 ± 1.63 ^d,e^	31.73 ± 1.39 ^d,e^	65.94 ± 2.95 ^g^
TL−80E	40.56 ± 1.68 ^f^	29.47 ± 1.48 ^d^	52.72 ± 2.84 ^e^
TL−C−W	36.37 ± 1.56 ^d^	28.24 ± 1.57 ^d^	57.85 ± 2.73 ^f^
TL−C−60E	26.59 ± 1.42 ^c^	18.53 ± 1.23 ^c^	39.29 ± 1.77 ^d^
TL−C−80E	25.18 ± 1.26 ^c^	20.58 ± 1.06 ^c^	33.89 ± 1.85 ^c^
Rosmarinic acid	16.72 ± 0.92 ^b^	8.34 ± 0.45 ^b^	30.64 ± 1.32 ^b^
Curcumin	9.14 ± 0.68 ^a^	ND	ND
L-ascorbic acid	ND	6.91 ± 0.26 ^a^	ND
α-Tocopherol	ND	ND	14.32 ± 0.93 ^a^

All values are expressed as mean ± standard deviation (*n =* 3). Different letters in each tested method indicate a significant difference (*p* < 0.05). ND: not determined.

## Data Availability

The original contributions generated for this study are included in the article; the data presented in this study are available on request from the corresponding author.
